# Habitual physical activity mediates the acute exercise-induced modulation of anxiety-related amygdala functional connectivity

**DOI:** 10.1038/s41598-019-56226-z

**Published:** 2019-12-24

**Authors:** Yu-Chun Chen, Chenyi Chen, Róger Marcelo Martínez, Jennifer L. Etnier, Yawei Cheng

**Affiliations:** 1 Department of Physical Medicine and Rehabilitation, National Yang-Ming University Hospital, Yilan, Taiwan; 2 Institute of Neuroscience and Brain Research Center, National Yang-Ming University, Taipei, Taiwan; 3 Graduate Institute of Injury Prevention and Control, College of Public Health, Taipei Medical University, Taipei, Taiwan; 4 Research Center of Brain and Consciousness, Shuang-Ho Hospital, Taipei Medical University, New Taipei City, Taiwan; 5 Graduate Institute of Mind, Brain and Consciousness, College of Humanities and Social Sciences, Taipei Medical University, Taipei, Taiwan; 6 Department of Kinesiology, University of North Carolina at Greensboro, Greensboro, NC USA; 7 Department of Research and Education, Taipei City Hospital, Taipei, Taiwan; 8 Cell Physiology and Molecular Image Research Center, Wan Fang Hospital, Taipei Medical University, Taipei, Taiwan

**Keywords:** Neuroscience, Amygdala

## Abstract

Aerobic exercise, in relation to physical activity, has been shown to have beneficial effects on anxiety. However, the underlyig neural mechanism remains elusive. Using a within-subject crossover design, this fMRI study examined how exercise (12-min treadmill running versus walking) mediated amygdala reactivity to explicit and implicit (backward masked) perception of emotional faces in young adults (*N* = 40). Results showed that acute exercise-induced differences of state anxiety (STAI-S) varied as a function of individual’s habitual physical activity (IPAQ). Subjects with high IPAQ levels showed significant STAI-S reduction (*P* < 0.05). Path analyses indicated that IPAQ explained 14.67% of the variance in acute exercise-induced STAI-S differences. Running elicited stronger amygdala reactivity to implicit happiness than fear, whereas walking did the opposite. The exercise-induced amygdala reactivity to explicit fear was associated with the IPAQ scores and STAI-S differences. Moreover, after running, the amygdala exhibited a positive functional connectivity with the orbitofrontal cortex and insula to implicit happiness, but a negative connectivity with the parahippocampus and subgenual cingulate to implicit fear. The findings suggest that habitual physical activity could mediate acute exercise-induced anxiolytic effects in regards to amygdala reactivity, and help establish exercise training as a form of anxiolytic therapy towards clinical applications.

## Introduction

The general consensus holds that exercise has beneficial effects on anxiety, as it has been observed that individuals who regularly engage in physical activity experience fewer anxiety symptoms^1^. While the effects of exercise and physical activity on cognitive functioning are well established, the neural mechanisms underlying their impact on emotional processing remain relatively elusive.

Importantly, physical activity, exercise, and physical fitness describe different yet closely intertwined concepts, where each of them may interactively contribute to the beneficial effects on anxiety^2^. A substantial body of literature suggests that physical activity has positive influences on anxiety^1,3–5^. When it comes to exercise (defined as a subset of planned, structured, and repetitive physical activity that has a final or an intermediate goal for the improvement or maintenance of physical fitness), acute and chronic exercise (a single bout vs. several consecutive weeks of sustained performance, respectively) can exert certain effects on anxiety relief  ^6–11^. One meta-analysis with healthy subjects reported that acute bouts of exercise yielded a small reduction in state anxiety by approximately 1/4 standard deviation^12^. Another meta-analysis in patients with anxiety and/or stress-related disorders indicated that exercise significantly decreased anxiety symptoms with a moderate effect size^13^. Nevertheless, these studies’ assessments of anxiety were dominated by self-reported measures. Only a few studies have used a combination of self-reported measures with electroencephalography (EEG) and electromyography (EMG) to examine the anxiolytic effects of acute exercise^14–16^. Moderate-intensity cycling exercise altered emotional arousal and hemispheric asymmetry in the beta frequencies to unpleasant stimuli, indicative of decreased EEG activity^15^. Resting frontal EEG asymmetry predicted positive affect following aerobic exercise on a treadmill^14^. Self-reported anxiety along with baseline corrugator supercilii EMG responses were reduced after cycling^16^.

A key brain structure implicated in anxiety is the amygdala, which is responsible for the detection of salient environmental features including threats^17^. As such, the magnitude of amygdala reactivity in response to threatening stimuli (anger and fear) has been associated with anxiety^18–20^. Given fearful faces are more ambiguous, neuroimaging research typically indicates that the amygdala tends to be more reactive to fearful than angry faces^21^. That is, fearful faces signify the presence of danger, but do not provide information about its source. Consequently, the threat-processing system is more reactive to ambiguous (or indirect) threat cues, as it has evolved to promote attention in order to determine appropriate responses (e.g., escape or approach)^22^. Because of this, amygdala reactivity to fearful faces is a reliable way of probing the neural correlates underlying anxiety^23–25^. Furthermore, emotional responses involve a complex and continuous interplay of explicit and implicit processes^26,27^. Non-masked stimuli consisted of displaying faces for 200-ms, while backwardly masked stimuli consisted of 17-ms of emotional faces followed by 183-ms of neutral faces. Using fMRI in conjunction with backwardly masked stimulus presentation could determine amygdala reactivity to implicit processing^28^. The implicit or non-conscious perception has been used to refer to a perceptual state in which the subjects do not report the presence of a stimulus, even though the stimulus has in fact been processed^27^.

Given that the anxiolytic effect of exercise might vary across individuals in relation with their lifestyles^12,14,29,30^, we assessed habitual physical activity by using the International Physical Activity Questionnaire (IPAQ), in addition to assessing anxiety with the State-Trait Anxiety inventory (STAI). It is reasonable to propose that exercise would alter responses implicated in low-level affective arousal (such as amygdala reactivity), all through brain circuits involved in executive control depending on emotional valence, which, in turn, could achieve anxiety relief. Here, this fMRI study used a backward masking paradigm, and adopted a within-subject crossover design to test whether acute aerobic exercise would alter amygdala reactivity and functional connectivity in response to explicit and implicit emotional processing. Moreover, we applied path analyses to corroborate the relationship among acute exercise-induced anxiolytic effect, habitual physical activity, and physical fitness.

## Materials and Methods

### Subjects

Forty healthy volunteers, aged between 20 to 30 (23.9 ± 2.4) years, were recruited using an online survey disseminated through social media. Because of poor fMRI quality ascribed to excessive head movement, one participant was excluded, resulting in a total of 39 subjects (15 females) in the data analysis. All subjects were prescreened to exclude comorbid psychiatry/neurological disorders (e.g., dementia, seizures), history of head injury, and alcohol or substance abuse or dependence within the past five years. All participants had normal or corrected-to-normal visual acuity and were free of medication at the time of the experiment. Each participant received instructions regarding all the experimental details, as well as their right to withdraw from the study at any time, and proceeded to sign an informed consent form. This study was approved by the Ethics Committee of National Yang-Ming University and conducted in accordance with the Declaration of Helsinki. In addition, subjects refrained from caffeine and alcohol intake for 24 hours before the experiment and during the experimental days.

### Stimuli

The visual stimuli used during fMRI scanning consisted of black and white pictures of male and female faces with happy, fearful, and neutral facial expressions, which were chosen from the Pictures of Facial Affect^31^. Faces were cropped into an elliptical shape with the background, hair, and jewelry cues eliminated, and were oriented as to maximize inter-stimulus alignment of the eyes and mouth. Faces were then artificially colored (red, yellow, or blue) and equalized for luminosity.

### Procedures

The experiment used a within-subject crossover design (Supplementary Fig. s1). The order of the running and walking sessions was counter-balanced between subjects. Half of the subjects first performed the running session, and the other half first performed the walking session. We randomly assigned subjects to the two different experimental sequences. Each session included a 3-min warm up, a 12-min running or walking session, and a 10-min cool down, followed with a 20-min fMRI scanning. As previous studies, including meta-analyses, suggested that positive affective responses would be experienced after relatively short bouts of acute exercise^10,12,32,33^, we determined to have a 12-min running with moderate to vigorous intensity for acute exercise. The cool down included a 5-min walking cool-down and a 5-min seated recovery.

To avoid carryover effect, there was at least a one-week interval between the running and walking sessions^34^. We did screen the participants’ significant life events during the past week and the day before either the running or walking sessions, to ensure that there were no significant events that could have an impact on their baseline anxiety levels. We also considered diurnal mood variation. If participants came to the experiment in the morning during one session, then they would come to the other session also in the morning (afternoon to afternoon).

#### Habitual physical activity

At the beginning of the experiment, subjects completed the International Physical Activity Questionnaire (IPAQ), which is a standardized self-report measure for habitual physical activity^35,36^. The IPAQ has three categories (levels) of physical activity^37^. Category 1 (low) refers to individuals who do not meet the criteria for categories 2 or 3, thus they are considered inactive and have the lowest level of physical activity. Category 2 (moderate) meets any one of the following three criteria: a) 3 or more days of vigorous activity of at least twenty minutes per day; b) 5 days or more of moderate-intensity activity or walking of at least thirty minutes per day; or c) 5 days or more of any combination of walking, moderate- or vigorous-intensity activities achieving a minimum of at least 600 estimated Metabolic Equivalent Task minutes per week (MET-min/week). Category 3 (high) meets any one of the following two criteria: a) vigorous-intensity activity on at least 3 days and accumulating at least 1500 MET-min/week; or b) 7 days or more of any combination of walking, moderate- or vigorous-intensity activities achieving a minimum of at least 3000 MET-min/week.

#### Acute exercise

At first, the resting heart rate (HRrest) was measured when the subjects were instructed to sit quietly for 5 min. The predicted maximum heart rate (HRmax) was determined by subtracting the participant’s age from 220. The volume of maximum oxygen consumption (estimated VO_2_max) ml · kg^−1^ · min^−1^ was estimated by HRmax/HRrest x 15.3 ml (kg.min)^38–40^. In the running session, subjects ran on a treadmill with a 0% incline and a 3-min warm up at subject’s self-selected speed, followed with a 12-min running when subjects were instructed to maintain a speed so that their HR was in the range of 64% to 96% HRmax. HR was continuously recorded using a Polar HR monitor (Epson RUNSENSE SF-810V). According to Borg’s rating of perceived exertion (RPE) scale^41^, subjects reported their perceived exertion on a 15-point scale (6 to 20) during exercise every two minutes. The scale ranges in description from 6 (no exertion at all) to 20 (maximal exertion).

In the walking session, subjects walked on a treadmill with a 0% incline for a duration of 12 min at the controlled distance of 0.39 ± 0.01 (0.38 ~ 0.40) km and limited speed of 1.95 ± 0.03 (1.90 ~ 2.05) km/hr. The walking session was set as a comparison condition that differed from the experimental condition (running session) only in the volitional exertion required to walk at light intensity.

#### Self-reported anxiety

The State-Trait Anxiety inventory (STAI) was administered to determine the self-reported anxiety levels of the subjects^42^. The STAI consists of two twenty-question scales, one to measure state anxiety (STAI-S), and one to measure trait anxiety (STAI-T). Each question is scored on a 4-point Likert-type scale, ranging from 1 to 4 (from “not at all” to “very much so” for the STAI-S, and from “almost never” to “almost always” for the STAI-T), with a total score range of 20 to 80. STAI-S verifies anxiety in specific situations; STAI-T confirms anxiety as a general trait. At the beginning of the experiment, subjects filled in STAI-T. Given that the top range of the STAI-T scores might suggest subjects with unreported anxiety disorders, we used a structural clinical interview tool to ensure all subjects exhibited no evidence of anxiety disorders^43^. After a 10-min cool down following acute exercise, subjects filled in STAI-S.

### fMRI scanning

The fMRI scanning paradigm was derived from the work by Etkin *et al*.^18^. Each stimulus presentation included a 200-ms fixation period to cue subjects to focus on the center of the screen, followed by a 400-ms blank screen and 200-ms of face presentation. Subjects were then allowed 1200-ms to respond by pressing a key indicating the color of the face. Non-masked stimuli consisted of displaying faces for 200-ms with an emotional (fearful or happy) or neutral expression face, while backwardly masked stimuli consisted of 17-ms of an emotional or neutral face, followed by 183-ms of a neutral face mask belonging to a different individual, but of the same color and gender as the previous one. Each ON block (12-s) consisted of six trials of the same stimulus type but randomized with respect to color and gender. A total of 12 ON blocks (two for each stimulus type) and 12 OFF blocks (a 12-s fixation cross) were pseudo-randomized for the stimulus type. To avoid stimulus order effects, we used two different counterbalanced run orders. During fMRI scanning, subjects performed the color identification task, in which they were asked to judge the color of each face (pseudo-colored in either red, yellow, or blue) and to indicate the answer by a keypad button press. The averaged reaction time was determined only for trials where subjects correctly identified the color of the faces (0.34 ± 0.09 sec). The average accuracy (±SEM) for all stimuli was 96 ± 0.1%.

Stimuli were presented using MATLAB software (MathWorks, Inc., Sherborn, MA, USA) and were triggered by the first radio frequency pulse for the functional run. The stimuli were displayed on VisuaStim XGA LCD screen goggles (Resonance Technology, Northridge, CA). The screen resolution was 800 × 600 with a refresh rate of 60 Hz. Behavioral responses were recorded by the fORP interface unit and saved in Matlab. Prior to the functional run, subjects were trained in the color identification task using unrelated face stimuli that were cropped, colorized, and presented in the same manner as the nonmasked faces described above, as to avoid any learning effects during the functional run.

Immediately after fMRI scanning, subjects underwent the detection task, during which subjects were shown all the stimuli again, and alerted of the presence of emotional (fearful, happy, or neutral) faces. Subjects were administered a forced-choice test under the same presentation conditions as during scanning and asked to indicate which emotional face they observed. The detection task was designed to assess possible awareness of the masked emotional faces. The chance level for correct answers was 33.3%. Better than chance performance was determined by calculating a detection sensitivity index (*ď*) based upon the percentage of trials in which a masked stimulus was detected when presented [‘hits’ (H)] adjusted for the percentage of trials a masked stimulus was ‘detected’ when not presented [‘false alarms’ (FA)]; [*d*′ = z-score (percentage H) − z-score (percentage FA), with chance performance = 0 ± 1.74]^44^. Each participant’s detection sensitivity was calculated separately for each of the stimulus categories and then averaged.

### fMRI data acquisition, image processing and analysis

Functional and structural MRI data were acquired on a 3 T MRI scanner (Siemens Magnetom Tim Trio, Erlanger, German) equipped with a high-resolution 32-channel head array coil. A gradient-echo, T2*-weighted echoplanar imaging (EPI) with a blood oxygen level-dependent (BOLD) contrast pulse sequence was used for functional data. To optimize the BOLD signal in the amygdala^45^, twenty-nine interleaved slices were acquired along the AC–PC plane, with a 96 × 128 matrix, 19.2 × 25.6 cm^2^ field of view (FOV) and 2 × 2 × 2 mm voxel size, resulting in a total of 144 volumes for the functional run (TR = 2 s, TE = 36 ms, flip angle = 70°, slice thickness 2 mm, no gap). Parallel imaging GRAPPA with factor 2 was used to increase the speed of acquisition. Structural data were acquired using a magnetization-prepared rapid gradient echo sequence (TR = 2.53 s, TE = 3.03 ms, FOV = 256 × 224 mm^2^, flip angle = 7°, matrix = 224 × 256, voxel size = 1.0 × 1.0 × 1.0 mm^3^, 192 sagittal slices/slab, slice thickness = 1 mm, no gap).

Image processing and analysis were performed using SPM8 (Wellcome Department of Imaging Neuroscience, London, UK) in MATLAB 7.0 (MathWorks Inc., Sherborn, MA, USA). Structural scans were coregistered to the SPM8 T1 template, and a skull-stripped image was created from the segmented gray matter, white matter, and CSF images. These segmented images were combined to create a subject-specific brain template. EPI images were realigned and filtered (128-s cutoff), then coregistered to these brain templates, normalized to MNI space, and smoothed (4 mm FWHM). The voxel size used in the functional analysis was 2 × 2 × 2 mm^3^. All subjects who completed scanning had less than 1 voxel of in-plane motion. A two-level approach for block-design fMRI data was adopted, using general linear model implemented in SPM8. Fixed effects analyses were performed at the single subject level to generate individual contrast maps and random effects analyses were performed at the group level. At the single subject level, contrast images were calculated comparing each explicitly and implicitly presented face block (happy and fearful) with the neutral baseline. These contrast images were then entered to the second level group analysis. The resulting first-level contrast images were then entered into analysis of variance (ANOVA): 2 (session: running vs. walking) × 2 (attention: explicit vs. implicit) × 2 (emotion: happiness vs. fear). Whole brain activations were corrected for multiple comparisons family-wise error (FWE) rate at *P* < 0.05.

Using MarsBar (see http://marsbar.sourceforge.net/), regions of interest (ROIs) were drawn from the right and left amygdala according to a prior study^46^. Given inconsistent laterality during emotion face recognition^47^, amygdala reactivity was determined by the averaged activity of right and left amygdala. Signals across all voxels within a radius of 4 mm in these ROIs were averaged and evaluated for the masked and nonmasked emotional (fearful and happy) comparisons. Shorthand (e.g., EF–EN) was used to indicate the contrasts of regressors (e.g., explicit fearful blocks > explicit neutral blocks). Error bars signify standard error of the mean. To isolate the effects of the emotional content of stimuli from other aspects of the stimuli and the task, we subtracted neutral (EN) or masked neutral (IN) activity from emotional (EF, EH) or masked emotional (fearful and happy) activity (IF, IH), respectively. The explicit perception of fearful and happy faces was denoted as nonmasked fear and happiness (EF–EN, EH–EN) and the implicit perception of fearful and happy faces as masked fear and happiness (IF–IN, IH–IN).

### Functional connectivity analysis

Based on our ROI results and prior study^18^, the psychophysiological interaction (PPI) analysis was seeded in the right amygdala (26, −10, −12; 20, −7, −15) to estimate how a single bout of aerobic exercise altered functional connectivity of the amygdala in response to implicitly perceived fearful and happy faces. The time series of the first eigenvariates of the BOLD signal were temporally filtered, mean corrected, and deconvolved to generate the time series of the neuronal signal for the source region, i.e., the amygdala, as the physiological variable in the PPI. PPI analysis assesses the hypothesis that the activity in one brain region can be explained by an interaction between cognitive processes and hemodynamic activity in another brain region. As the amygdala was selected as the PPI source region, the physiological regressor was denoted by the activity in the amygdala. The implicit (fearful or happy) condition was the psychological regressor. The interaction between the first and second regressors represented the third regressor. The psychological variable was used as a vector coding for the specific task (1 for running, −1 for walking) convolved with the hemodynamic response function. The individual time series of the amygdala was obtained by extracting the first principle component from all raw voxel time series in a sphere (4 mm radius) centered on the coordinates of the subject-specific amygdala activations. These time series were mean-corrected and high-pass filtered to remove low-frequency signal drifts. The physiological factor was then multiplied by the psychological factor to constitute the interaction term. PPI analyses were then carried out for each subject by creating a design matrix with the interaction term, the psychological factor, and the physiological factor as regressors. Subject-specific contrast images were then entered into random effects analyses (thresholded at *P* < 0.01, uncorrected, *k* = 10). PPI analyses were performed for each session separately (running and walking) to identify brain regions showing significant differences in functional coupling with the amygdala during implicitly perceived fear and happiness in relation to a single bout of aerobic exercise. In order to identify brain regions showing significant differences in functional coupling with the amygdala during implicit fear and happiness in relation to a single bout of aerobic exercise, subject-specific contrast images were then entered into random effects analyses (thresholded at *P* < 0.01, uncorrected, *k* = 20).

### Statistical analysis

The software SPSS 17.0 and IBM SPSS AMOS 23.0 were used for statistical analyses. Path analysis was performed to examine the relationships and directionality among habitual physical activity, acute exercise-induced anxiolytic effect, and physical fitness (Supplementary Fig. s2). Eight models (2^3^) with three observed variables (IPAQ, ΔSTAI-S, VO_2_max) and eight optional neighboring paths (2^3^) were tested using Bayesian Information Criterion (BIC)^48^, which entailed quantifying model evidence (favoring fit accuracy and penalizing complexity). The estimating coupling parameters were reported. The optimally fitting model should fulfill the criteria of a χ^2^ statistic corresponding to *P* > 0.05 and a standardized root mean square residual value < 0.08. C is the discrepancy function (which in this case is the likelihood ratio chi-square statistic), and smaller values of the discrepancy function indicate a more favorable fit of the model to data. The Bayesian Information Criterion (BIC) is a criterion for model selection from among a finite set of models (the model with the lowest BIC is preferred). It is partly based on the likelihood function and is closely related to the Akaike Information Criterion (AIC). An AIC value spanning from 2 to 4 is definite evidence that the model should be ruled out as being the actual Kullback−Leibler optimal model for the population of possible samples.

## Results

### Behavioral performance

Estimated VO_2_max ranged from 36.96 to 52.67 with mean and standard deviation 43.24 ± 3.15 ml · kg^−1^ · min^−1^. In the running session, HR (% HRmax) and RPE were 140.3 ± 10.0 beats per minute (71 ± 5.0%) and 12.7 ± 0.9 points, respectively, which it falls in the range of moderate- and vigorous-intensity exercise according to the Guidelines of the American College of Sports Medicine (ACSM)^49^. In the walking session, HR (% HRmax) and RPE were 75.2 ± 5.3 beats per minute (39 ± 5.1%) and 9.6 ± 0.8 points, respectively. HR (% HRmax) and RPE were significantly different between the running and walking sessions (all *P* < 0.001).

The STAI assessments indicated that the mean (SD) and median of trait anxiety (STAI-T) showed 41.3 (9.0) and 38.0 with a range of 27 to 65. State anxiety (STAI-S) did not significantly differ between the running and walking sessions (32.1 ± 7.7 vs. 32.9 ± 8.3; *t*_38_ = 0.71, *P* = 0.348). As for their IPAQ scores, subjects corresponding to category 1 (*n* = 5), 2 (*n* = 24), and 3 (*n* = 9) had low, moderate, and high levels of habitual physical activity, respectively (mean ± SD: 322.2 ± 75.48 vs. 1782.21 ± 124.48 vs. 4545.56 ± 282.27 MET-min/week). To address the raised concerns about if individuals who exercised more regularly might select more intense exercise intensity, we found that the groups with differential levels of habitual physical activity (low vs. moderate vs. high IPAQ scores) did not significantly differ in the distance (*F*_2, 35_ = 1.46, *P* = 0.25), HRrest (*F*_2, 35_ = 1.46, *P* = 0.25), HR (*F*_2, 35_ = 0.52, *P* = 0.60), and RPE (*F*_2, 35_ = 1.08, *P* = 0.37) during the running session.

**Figure 1:**
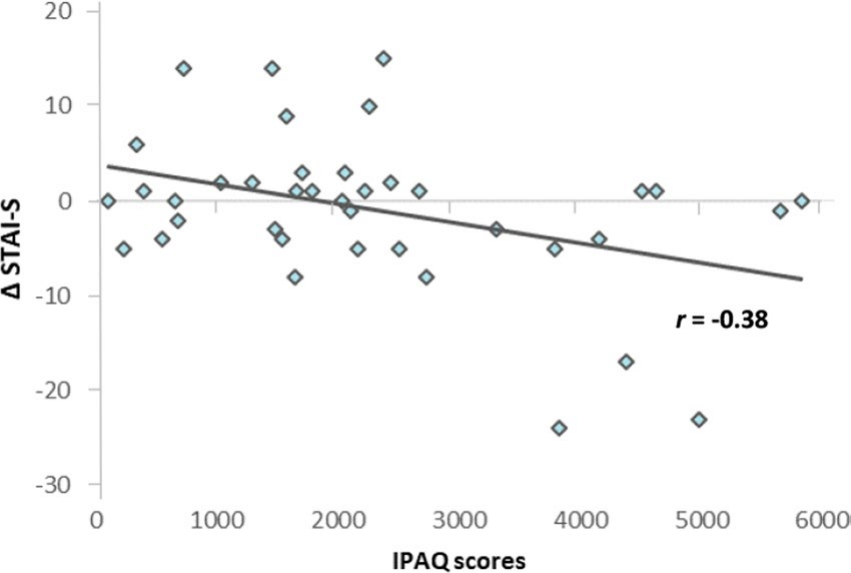
The relationship between acute exercise-induced anxiolytic effect and habitual physical activity. Acute exercise-induced anxiolytic effect, as indicated by the running vs. walking STAI-S differences (ΔSTAI-S), varies as a function of the individual habitual physical activity, as assessed by the IPAQ (*r* = −0.38, *P* = 0.015).

Interestingly, acute exercise-induced anxiolytic effect, as indicated by the running vs. walking STAI-S differences (ΔSTAI-S), varied as a function of the individual habitual physical activity, as assessed by the IPAQ (*r* = −0.38, *P* = 0.015) (Fig. 1). The relationship of ΔSTAI-S and IPAQ remained significant even after controlling for STAI-T [β = −0.47, *P* = 0.003, R^2^ = 0.187, (*F*_2, 34_ = 5.15, *P* = 0.011)] and adjusting for estimated VO_2_max and measurement error [β = −0.46, *P* = 0.004, R^2^ = 0.156, (*F*_2, 34_ = 4.65, *P* = 0.016)]. The magnitude of the association between ΔSTAI-S and IPAQ was twenty times greater than that between ΔSTAI-S and estimated VO_2_max [β = 0.015, *P* > 0.9, R^2^ = 0.169, (*F*_2, 34_ = 24.65, *P* = 0.016)].

For the color identification task during fMRI scanning, the accuracy did not differ between explicit and implicit conditions (*t*_38_ = 1.07, *P* = 0.304) and between running and walking sessions (32.1 ± 7.7 vs. 33.0 ± 8.3; *t*_38_ < 0.01, *P* = 0.99). For the detection task outside the fMRI scanner, according to a one-tailed binominal model, a score of 28 hits (42.2% hit rate) or above was considered significantly over chance level (33%). All subjects performed above chance in the explicit condition (*d*’, mean ± SD: 2.80 ± 1.42) but below chance in the implicit condition (0.04 ± 0.06). The number of hits was not significantly different between the running and walking sessions regardless of the explicit (*t*_38_ = −0.59, *P* > 0.05) or implicit condition (*t*_38_ = 0.91, *P* > 0.05).

### Path analysis results

**Figure 2:**
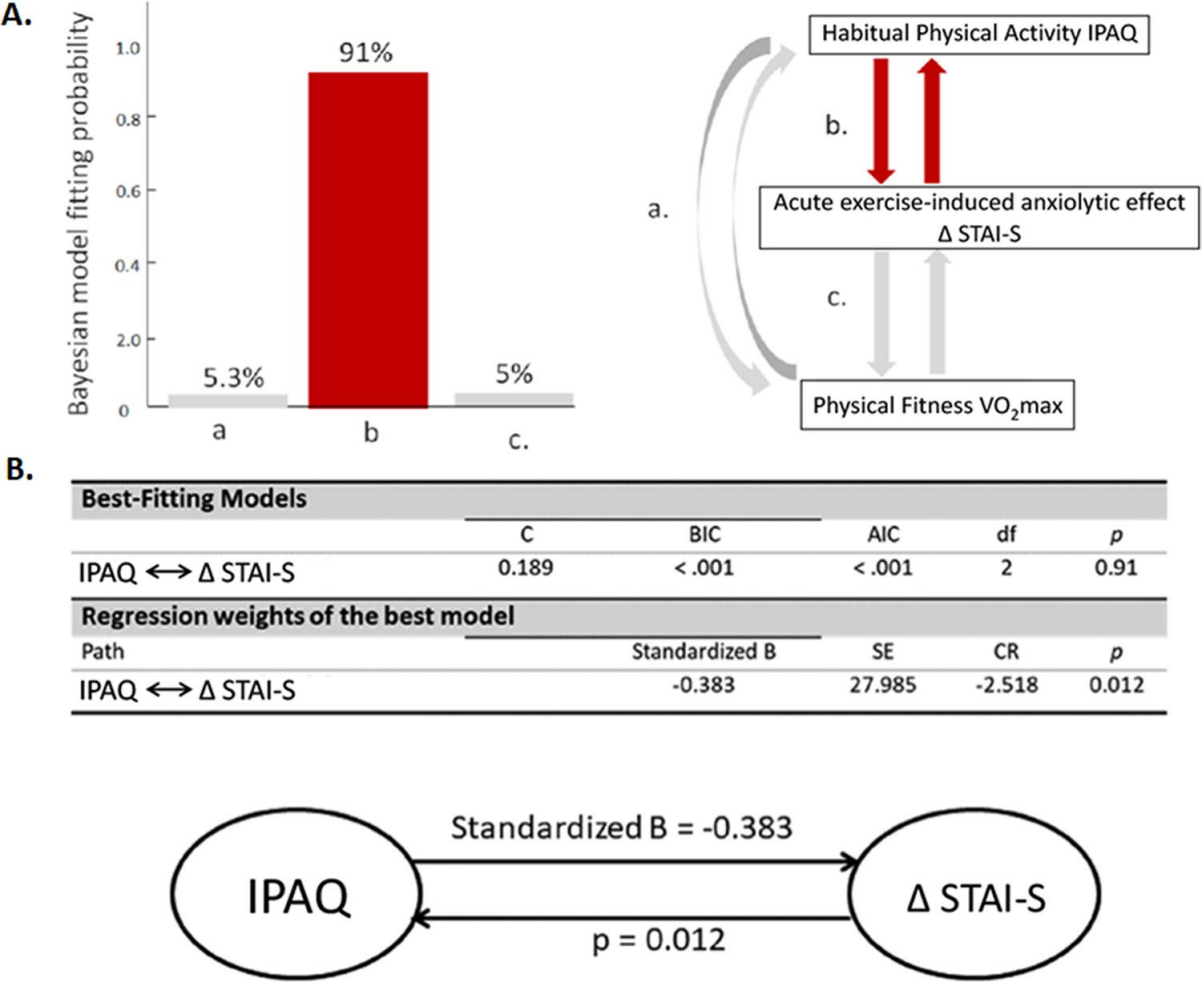
The best-fitting probability of hypothetical models for the relationships and directionality among three variables of habitual physical activity IPAQ, acute exercise-induced anxiolytic effect ΔSTAI-S, and physical fitness VO_2_max). (**A**) The model b of IPAQ ↔ΔSTAI-S exhibited the highest model fitting probability (91%) among eight models (2^3^) with three observed variables (IPAQ, ΔSTAI-S, VO_2_max) and eight optional neighboring paths (2^3^). (**B**) The regression weight estimate is 2.518 standard errors below zero. The standardized path coefficients (SE) indicate that the IPAQ explains 14.67% of the variance in Δ STAI-S. Abbreviations: df, degree of freedom; B, estimated beta value; SE, standard error; CR, critical ratio obtained by dividing the covariance estimate by its standard error.

With structural equation modeling, a model comparison strategy that balances the trade-off between model goodness of fit and model complexity was used to identify the optimal model among alternatives. The model of IPAQ ↔ ΔSTAI-S exhibited the highest model fitting probability (91%) among eight models with three observed variables (IPAQ, ΔSTAI-S, VO_2_max) and eight optional neighboring paths. The discrepancy function, BIC index, AIC value, degrees of freedom, and model fitting probability of the best-fitting model are listed in Fig. 2. The standardized path coefficients (SE) for the optimally fitting model showed that the IPAQ could explain 14.67% of the variance in ΔSTAI-S.

### fMRI data

The whole-brain analysis showed significant hemodynamic differences after a single bout of aerobic exercise during explicit and implicit emotional processing (Table 1). During explicit fear (EF–EN), running as compared with walking significantly increased the hemodynamic response in the fusiform gyrus. Walking relative to running increased the responses in the amygdala, inferior frontal gyrus, and primary visual cortex. During explicit happiness (EH–EN), running relative to walking significantly reduced the responses in the amygdala, middle frontal gyrus, middle occipital gyrus, and superior temporal gyrus.

**Table 1:** Brain regions showing significant hemodynamic responses to emotional processing after acute exercise.

Brain Region	side	MNI Coordinates			*t*-value	*k*
		x	y	z		
**Run vs. Walk|Explicit Fear (EF–EN)**						
Fusiform gyrus	L	−36	−42	−14	3.87	19
**Walk vs. Run|Explicit Fear (EF–EN)**						
Amygdala	L	−26	−8	−12	2.21*	2
Inferior frontal gyrus	R	−22	32	−8	3.18	20
Primary visual cortex	R	−14	−88	0	3.16	7
**Run vs. Walk|Implicit Fear (IF–IN)**						
Parahippocampus	R	24	−58	−10	3.48	6
Thalamus	R	6	−22	0	3.67	10
**Walk vs. Run|Implicit Fear (IF–IN)**						
N.S.						
**Run vs. Walk|Explicit Happiness (EH–EN)**						
N.S.						
**Walk vs. Run|Explicit Happiness (EH–EN)**						
Amygdala	L	−26	−2	−18	2.08*	5
Middle frontal gyrus	L	−20	32	−8	4.91	26
Middle occipital gyrus	R	38	−84	−4	3.82	20
Superior temporal gyrus	R	48	−24	0	3.71	5
**Run vs. Walk|Implicit Happiness (IH–IN)**						
Amygdala	L	−22	−5	−16	2.47*	19
Amygdala	R	24	−6	−15	2.35*	18
Inferior frontal gyrus	L	−36	26	−10	3.87	11
Lateral occipital gyrus	R	18	−72	−8	4.21	28
**Walk vs. Run|Implicit Happiness (IH–IN)**						
Superior temporal gyrus	L	−52	−8	−8	3.12	5

Pooled group results for all subjects (*n* = 39). All clusters are significant at FWE-corrected *P* < 0.05, and only clusters of 10 or more contiguous voxels are reported, except those marked with an asterisk, which are taken from predefined ROIs and significant at uncorrected *P* < 0.05. Abbreviations: R, right; L, left; EF, explicit fear; EN, explicit neutral, IF, implicit fear; IN, implicit neutral; EH, explicit happiness; IH, implicit happiness.

During implicit fear (IF–IN), running relative to walking significantly increased the responses in the parahippocampus and thalamus. During implicit happiness (IH–IN), running relative to walking significantly increased the responses in the bilateral amygdala, inferior frontal gyrus, and occipital cortex, whereas significantly decreased the response in the superior temporal gyrus.

**Figure 3:**
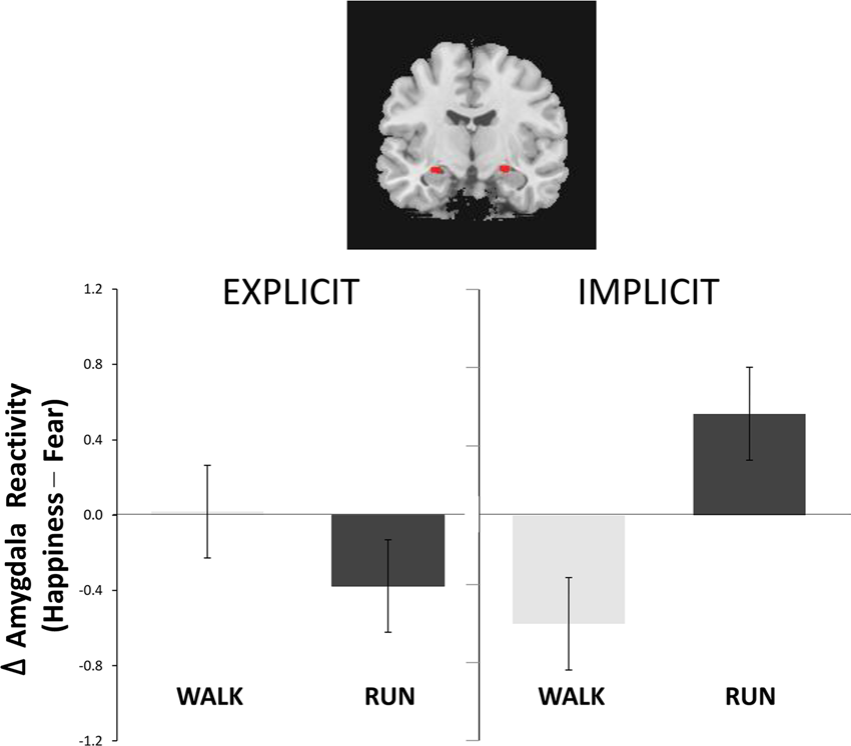
Amygdala reactivity after acute exercise. There was an interaction of session (running vs. walking) x attention (explicit vs. implicit) x emotion (happiness vs. fear) for amygdala reactivity (x 26, y −10, z −12; −26, −10, −12) (*F*_1, 38_ = 12.97, *P* = 0.001). *Post hoc* analyses indicated an interaction of emotion x session during the implicit condition (*F*_1, 38_ = 10.6, *P* = 0.002), but not during the explicit condition (*F*_1, 38_ = 1.74, *P* = 0.2). Follow-up tests showed that the running induced stronger amygdala reactivity to implicit happiness than to fear (*P* = 0.021), but the walking did the opposite (*P* = 0.013).

Regarding the ROI results, amygdala reactivity (x 26, y −10, z −12; −26, −10, −12) revealed an interaction between session (running vs. walking), attention (explicit vs. implicit), and emotion (happiness vs. fear) (*F*_1, 38_ = 12.97, *P* = 0.001, η^2^ = 0.25). The follow-up analyses indicated an interaction of emotion x session under the implicit condition (*F*_1, 38_ = 10.6, *P* = 0.002, η^2^ = 0.22), but none under the explicit condition (*F*_1, 38_ = 1.74, *P* = 0.2, η^2^ = 0.04). Tukey post hoc tests indicated that the emotion effect on amygdala reactivity had opposite directions depending on the factor of session. The running session had stronger amygdala reactivity to implicit happiness than fear (IH vs. IF: 0.141 ± 1.562 vs. −0.397 ± 1.210; *P = *0.021), whereas the walking session showed the opposite (−0.810 ± 1.225 vs. −0.233 ± 1.013; *P* = 0.013) (Fig. 3). Furthermore, we did not find any significant correlation of amygdala reactivity with IPAQ or estimated (all *r* < 0.80).

### Habitual physical activity, amygdala reactivity, and state anxiety

**Figure 4:**
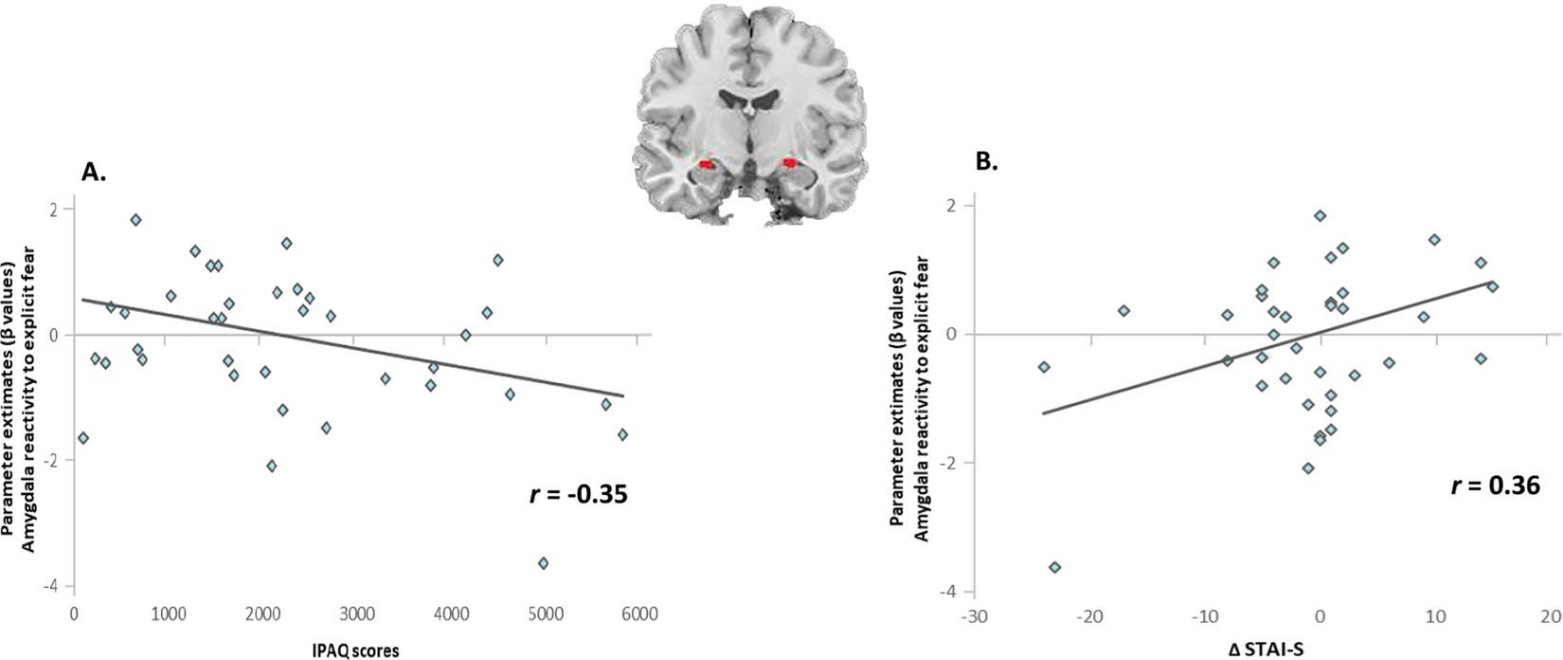
The relation between habitual physical activity, amygdala reactivity, and acute exercise-induced anxiolytic effect. (**A**) The exercise-induced amygdala reactivity to explicit fear was correlated with the IPAQ scores (*r* = −0.35, *P* = 0.029). (**B**) The exercise-induced amygdala reactivity to explicit fear was correlated with ΔSTAI-S (*r* = 0.36, *P* = 0.026). Individuals with more exercise-induced amygdala reactivity reported more anxiety relief.

The exercise-induced amygdala reactivity to explicit fear was correlated with the IPAQ scores (*r* = −0.35, *P* = 0.029) (Fig. 4A). When accounting for the STAI-T, the relation between the IPAQ and amygdala reactivity remained significant [β = −0.36, *P* = 0.024, R^2^ = 0.131, (*F*_2, 35_ = 3.79, *P* = 0.032)]. The exercise-induced amygdala reactivity to explicit fear was correlated with ΔSTAI-S (*r* = 0.36, *P* = 0.026). Individuals with more exercise-induced amygdala reactivity reported more anxiety relief (Fig. 4B). However, after removing one outlier who had weaker amygdala reactivity (subject 11), the relation between ΔSTAI-S and amygdala reactivity became non-significant (*r* = 0.18, *P* = 0.3), suggesting that this relationship might be unduly influenced by the datum in the lower left of Fig. 4B.

To further examine the role of amygdala reactivity in the association between the IPAQ and ΔSTAI-S, we applied a moderated multiple regression analysis in a hierarchical fashion by examining the statistical difference between the model without and with an interaction variable. The interaction variable represented the interaction between the IPAQ and amygdala reactivity. Before introducing the interaction variable, the model explained 19.2% of the overall variance with a significant correlation between the IPAQ and ΔSTAI-S (*r* = −0.38, *P* = 0.018). After introducing the interaction variable, the explanatory power of the model improved (27.3%). Both of the IPAQ [β = −0.337, *P* = 0.037, R^2^ = 0.273, (*F*_2, 33_ = 7.57, *P* = 0.002)] and the interaction variable [β = 0.34, *P* = 0.036, R^2^ = 0.273, (*F*_2, 33_ = 7.57, *P* = 0.002)] were significantly correlated with ΔSTAI-S, suggesting that amygdala reactivity should be a moderator in the relationship between the IPAQ and ΔSTAI-S.

### Functional connectivity

**Figure 5:**
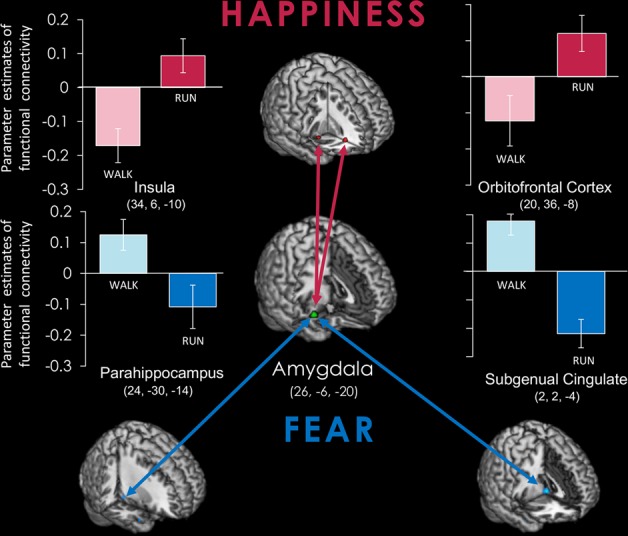
Functional connectivity of the amygdala after acute exercise. Running and walking trigger distinct patterns of functional connectivity to implicit emotional processing. Running relative to walking significantly upregulated the functional connectivity of the amygdala with the insula and the orbitofrontal cortex to implicit happiness, whereas it downregulated the functional connectivity of the amygdala with the subgenual cingulate and the parahippocampus to implicit fear.

Running and walking induced distinct patterns in the amygdala during emotional processing. To further examine the extent to which the acute exercise-induced modulation over low-level affective processing in the amygdala contributed to the functional coupling between brain regions, we subsequently assessed the connectivity in the amygdala where functions were related to the acute exercise effect. One bout of aerobic exercise significantly modulated the patterns of functional connectivity during implicit emotional processing (Fig. 5). After running relative to walking, implicit happiness induced a significantly positive connectivity of the amygdala with the orbitofrontal cortex (20, 36, −8) and insula (34, 6, −10). Implicit fear induced a significantly negative connectivity with the parahippocampus (24, −30, −14), subgenual cingulate (2, 2, −4), and fusiform gyrus (−54, −68, −4).

## Discussion

This fMRI study examined the neural mechanisms underlying the relation between the acute exercise-induced anxiolytic effect and habitual physical activity. Results showed that the acute exercise-induced anxiolytic effect, as indicated by ΔSTAI-S, varied as a function of the individual’s habitual physical activity, as assessed by the IPAQ. Path analyses corroborated this relation to probe the extent to which the IPAQ could explain the ΔSTAI-S variance. Running elicited stronger amygdala reactivity to implicit happiness than fear, whereas walking did the opposite. The exercise-induced amygdala reactivity to explicit fear was associated with the IPAQ and ΔSTAI-S. Furthermore, after running, the amygdala achieved a positive functional connectivity with the orbitofrontal cortex and insula to implicit happiness, but a negative connectivity with the parahippocampus and subgenual cingulate to implicit fear. The findings suggest that habitual physical activity could mediate acute exercise-induced anxiolytic effect in regards to amygdala reactivity.

### Habitual physical activity

After one bout of aerobic exercise, subjects with higher habitual physical activity reported more anxiety relief. The present findings appear in parallel with previous studies stating that individuals participating in regular exercise reported less anxiety and fatigue after acute bouts of aerobic exercise, whereas non-regular exercisers exhibited the opposite effect or no change at all^29,30^. Here, the performed path analysis further indicated that the IPAQ could explain 14.67% of the variance in ΔSTAI-S. Thus, to maximize positive mood changes following exercise, the individual’s habitual physical activity should be taken into account when prescribing exercise as a therapeutic tool.

Despite the fact that previous studies have indicated stable anxiety-reducing effects ascribed to acute exercise, the effect size varies across study designs and characteristics^6,10–13^. Among them, a within-subjects design yielded a larger effect size (*d* = 0.47) than the between-subject design with a no-treatment control group (*d* = 0.22). It is reasonable to infer that, without control groups, the absolute change in state anxiety might result from other uncontrolled factors, such as, testing effect, maturation, and instrumentation. Otherwise, introducing between-subject variables could inevitably bring in individual differences that are irrelevant and confounding to the study. One important individual variable that might significantly impact acute anxiolytic effects could be habitual physical activity at baseline. Even with randomized controlled trials (RCTs) that were originally designed for controlling between-subject variance, as well as for making causal inferences, the insufficient sample size still could not guarantee to have evenly distributed habitual physical activity in different treatment groups^50^. The findings regarding the acute exercise-induced anxiolytic effect could be inconsistent at best. Future studies considering a pseudo-randomized design to control individual differences in habitual physical activity are warranted.

In regards to the concept of design rationale, we utilized walking as the comparison condition. Previous research has mainly used the walking on a treadmill as the experimental condition, in particular with patients with anxiety and depression^51,52^. Nevertheless, and apart from the research probing into the anxiolytic effects of acute exercise, walking was more likely to be set as a control intervention in the RCTs^53,54^. Here, we probe the differences between treadmill running and walking in regards to self-reported anxiety, and amygdala reactivity, by taking the advantage of their differential exercise intensity and volitional exertion, as indicated by HR and RPE. Even though the identified effect in the present study may not be as strong as that uncovered by those prior using the quiet rest as control, ours still yielded significant results.

### Amygdala reactivity

Based on a within-subject crossover design with a counterbalanced order, we found that the amygdala reactivity achieved an interaction between session (running vs. walking), attention (explicit vs. implicit), and emotion (happiness vs. fear). Post hoc analyses indicated that the interaction of emotion by session existed for the implicit but not for the explicit condition. The interaction was mainly ascribed to the presence of significant differences between happy and fearful processing depending on the factor of walking or running. Interestingly, running elicited stronger amygdala reactivity to implicit happiness than fear, whereas walking had the opposite effect. Furthermore, despite of one identified outlier, a moderated multiple regression analysis indicated that amygdala reactivity could be a moderator in the relationship between the IPAQ and ΔSTAI-S. Given that prior knowledge regarding how the acute exercise-induced anxiolytic effect is represented and modulated by any underlying neural mechanism remains limited^14–16^, the present findings aid in broadening the literature concerning the relation between habitual physical activity, amygdala reactivity, and acute exercise-induced anxiolytic effect. The amygdala is sensitive to the valence of emotional stimuli^17^. Some studies have reported amygdala activation in response to emotionally negative photographs, whereas others have also reported amygdala activation to positive stimuli, which suggests that the amygdala responds more broadly to emotionally arousing and/or salient stimuli^55,56^. The increased amygdala sensitivity to implicit happiness could be explained by means of the mood congruency hypothesis, suggesting that amygdala reactivity and consequent shifts of attention should be correlated with mood-congruent stimuli^57,58^. Acute aerobic exercise significantly reduces cortisol responses during stressful tasks and thereby enhances positive affect^59^. This positive mood shifts might result in hypersensitivity to implicit perception of happy faces.

### Functional connectivity

Moreover, the PPI analysis indicated that running relative to walking upregulated the functional connectivity of the amygdala with the insula and the orbitofrontal cortex (OFC) to implicit happiness, whereas it downregulated the functional connectivity of the amygdala with the subgenual cingulate and the parahippocampus to implicit fear. The OFC is involved in the pursuit of happiness and of specific rewards^60^. The insula integrates disparate functional systems involved in cognition, sensory-motor, and affect processing^61^. The subgenual cingulate, which contributes to mood homeostasis and affective processing, has been found to be dysregulated in patients with anxiety disorders^62^. The functional connectivity between amygdala and parahippocampus could be activated during emotionally influenced memory storage^63^ and become aberrant in anxiety disorders^20^. The amygdala-centered resting-state functional connectivity was enhanced, indicating baseline hypervigilance that promotes saliency detection during the immediate aftermath of acute psychological stress and in patients with depression^64,65^. Here, we demonstrated that acute exercise would drive the amygdala-centered connectivity toward implicit happiness and against implicit fear. It lends support to the notion that exercise helps shift to a positive mood through not only regional functional modulation, but also the interacting brain networks. The exercise-induced negative functional connectivity between the amygdala, subgenual cingulate, and parahippocampus to negative emotion (fear) might form the neurobiological basis for exercise-induced anxiolytic effects. In parallel, one resting-state fMRI study in healthy sedentary volunteers found that aerobic exercise sustained for 16 weeks could reduce mood disturbances and strengthen the functional connectivity from the parahippocampus to regions involved in mood regulation^66^.

### Limitations

Some limitation of this study must be acknowledged. First, a lack of a gold standard (or submaximal) measure of cardiorespiratory fitness might be a potential limitation of this study. The formula for estimated VO2max was developed on highly trained individuals and so may not be precisely accurate in our sample. But the study is a within-subjects design, we are somewhat protected against any inaccuracies in this estimation formula. Second, regarding the timing for assessing state anxiety, STAI-S was only assessed after a 10-min cool down following acute exercise. We did screen to ensure that participants had no significant life events that could have an impact on their baseline anxiety levels during the experiment. We also took into account diurnal mood variation. Third, for the correlations of amygdala reactivity with IPAQ and ΔSTAI-S, we examined the scatterplots in detail. After the removal of the two outliers who had higher IPAQ scores (subject 7 and 16) in Figs. 1 and 4A, the relation between IPAQ and ∆STAI-S remained significant (*r* = −0.46, *P* = 0.004), and so was the relation between IPAQ and amygdala reactivity (*r* = 0.36, *P* = 0.026). Fourth, elevated heart rate, presumably after exercise, might confound neuroimaging results. There was a 10-min cool down between acute exercise and fMRI scanning. Given the variability in physical fitness, heart rate response would vary by participants and would need to be account for. Nevertheless, we do report the contrast within the conditions (running/happiness compared with running/fear, as well as walking/happiness compared to walking/fear), and which is a strength when it comes to this limitation. Fifth, lack of no-treatment control might be a major limitation of this study and future similarly designed studies would benefit from using three conditions (rest, running, walking). Finally, our sample is only young adults. This may not be the optimal design, and future studies are warranted with longitudinal researches in clinical populations or large community-based samples.

## Conclusions

Taken together, using a within-subject design and a backward masking paradigm for explicit and implicit emotional processing, this fMRI study analyzed the relationship between physical activity, acute exercise, and anxiety. Habitual physical activity could explain 14.67% of the variance in the acute exercise-induced anxiolytic effect, whose underlying neural mechanisms would be associated with amygdala reactivity and its functional connectivity. These findings allow the anxiolytic effect of exercise to be understood in terms of existing neurobiological knowledge, thereby providing support to the theory that exercise training recruits a process which confers enduring resilience to stress^67^. Importantly, this study helps guide future research towards enhancing the effectiveness of exercise training as treatment in clinical applications.

## Supplementary information

Supplementary Materials
