# Supplementary material for: Habitual physical activity mediates the acute exercise-induced modulation of anxiety-related amygdala functional connectivity
Source: Sci Rep. 2019 Dec 24;9:19787. doi: 10.1038/s41598-019-56226-z (PMC6930267; doi:10.1038/s41598-019-56226-z)

**Habitual physical activity mediates the acute exercise-induced modulation of anxiety-related amygdala functional connectivity**

Yu-Chun Chen^1,2^*, Chenyi Chen^3,4,5^*, Róger Marcelo Martínez^3,4,5^, Jennifer L. Etnier^6^, Yawei Cheng^1,2,7 CA^

1. Department of Physical Medicine and Rehabilitation, National Yang-Ming University Hospital, Yilan, Taiwan.

2. Institute of Neuroscience and Brain Research Center, National Yang-Ming University, Taipei, Taiwan.

3. Graduate Institute of Injury Prevention and Control, Taipei Medical University, Taipei, Taiwan.

4. Research Center of Brain and Consciousness, Shuang-Ho Hospital, Taipei Medical University, New Taipei City, Taiwan.

5.Institute of Humanities in Medicine, Taipei Medical University, Taipei, Taiwan.

6. Department of Kinesiology, University of North Carolina at Greensboro, NC, USA.

7. Department of Research and Education, Taipei City Hospital, Taipei, Taiwan.

* Equally contributed to the manuscript.

**Corresponding Author:** Prof. Yawei Cheng

Institute of Neuroscience and Brain Research Center

National Yang-Ming University

155, Sec. 2, St. Linong, Dist. Beitou, Taipei 112, Taiwan, ROC.

Tel: 886-2-28267912

Fax: 886-2-28264903

Email: [ywcheng2@ym.edu.tw](mailto:ywcheng2@ym.edu.tw)

**SUPPLEMENTARY MATERIALS**

**RESULTS**

Given that △STAI-S was modulated by the IPAQ, we further identified groups of low, moderate, and high IPAQ scores based on criterion definitions accordingly. We normalized the individual △STAI-S and performed a non-parametric paired-sample-*t* test (Wilcoxon signed rank tests) separately for each group. Results showed that △STAI-S reached significance in the group with high IPAQ (*n* = 9) (Z = -1.82, *P* =.034, one-tailed), but not in the groups with low (*n* = 5) (Z = -0.14, *P* = .89) and moderate IPAQ (*n* = 24) (Z = -1.52, *P* = .13).

Given that the amygdala reactivity was modulated by the IPAQ, we identified groups with low, moderate, and high IPAQ scores based on categorical definitions accordingly. We normalized the individual amygdala reactivity to explicit fear (EF–EN) and performed a non-parametric paired-sample-t test (Wilcoxon signed rank tests) separately for these groups. Results showed that the group with low IPAQ (*n* = 5) had no significance (Z = -0.41, *P* = .67), whereas the groups with moderate (*n* = 24) and high IPAQ (*n* = 9) showed significant exercise-induced amygdala reactivity to explicit fear (Z = -1.7, *P* = .045; Z = -1.72, *P* = .043 one-tailed).

**Figure s1: Experimental procedures.**

The experiment has a within-subject crossover design. Subjects first filled in the International Physical Activity Questionnaire (IPAQ) and trait anxiety (STAI-T) by State-Trait Anxiety inventory, then they proceeded to perform a running or walking condition. The order of running and resting sessions is counter-balanced between subjects, such that half of the subjects first perform the running session and the other half first perform the resting session. The subjects are randomly assigned to either of these two different experimental sequences. In the running session, subjects ran on a treadmill with a 0% incline and a 3-min warm up at subject's self-selected speed, followed with a 12-min running when subjects were instructed to maintain a speed so that their HR was in the range of 64% to 96% HRmax. In the walking session, subjects walked on a treadmill with a 0% incline for a duration of 12 min at the controlled distance and limited speed. They then had a 10-min cool down, after which they underwent a 20-min fMRI scanning. During the time interval between cool down and fMRI scanning, subjects filled in state anxiety (STAI-S). During fMRI scanning, subjects were asked to perform the color identification task. Immediately after fMRI scanning, the subjects did the detection task, which is designed to assess possible awareness of the masked emotional faces.

**
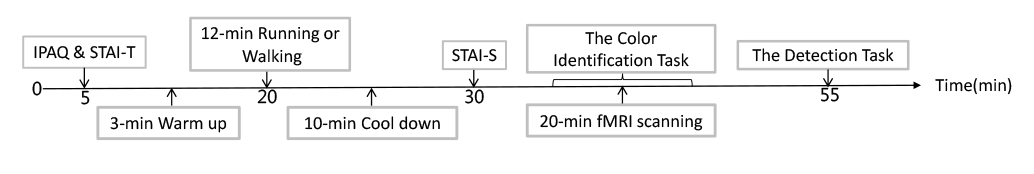
**

**Figure s2:** Hypothetical models for the relationships and directionality among IPAQ, △STAI-S, and VO2max.


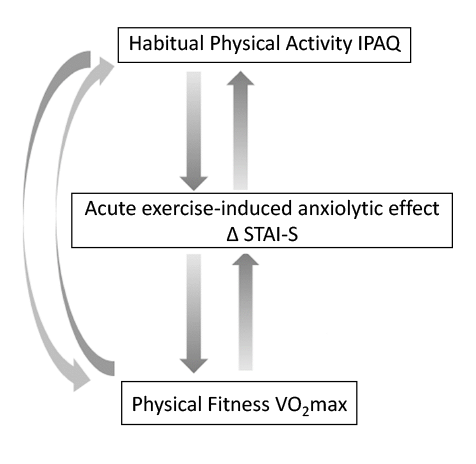


**Figure s3: Group-wise results of habitual physical activity and acute exercise-induced anxiolytic effect.** Given that △STAI-S was modulated by IPAQ, we further identified groups of low, moderate, and high IPAQ scores based on criterion definitions accordingly. We normalized the individual △STAI-S and performed a non-parametric one-sample-*t* test (Wilcoxon signed rank tests) separately for each group. Results showed that △STAI-S reached significance in the group with high IPAQ (*n* = 9) (Z = -1.82, *P* =.034, one-tailed), but not in the groups with low (*n* = 5) (Z = -0.14, *P* = .89) and moderate IPAQ (*n* = 24) (Z = -1.52, *P* = .13).

**A.** Acute exercise-induced anxiolytic effect, as indicated by the running vs. walking STAI-S differnces (△STAI-S), varies as a function of the individual habitual physical activity, as assessed by IPAQ (*r* = -0.38, *p* = .018).

**B.** After identifying groups with low, moderate, and high IPAQ scores, the individual △STAI-S was normalized and a non-parametric one-sample-t test (Wilcoxon signed rank tests) was performed separately for each group. △STAI-S reached significance only in the group with high IPAQ (*n* = 9) (Z = -1.82, *P* =.034, one-tailed), but not in the groups with low (*n* = 5) (Z = -0.14, *P* = .89) and moderate IPAQ (*n* = 24) (Z = -1.52, *P* = .13)


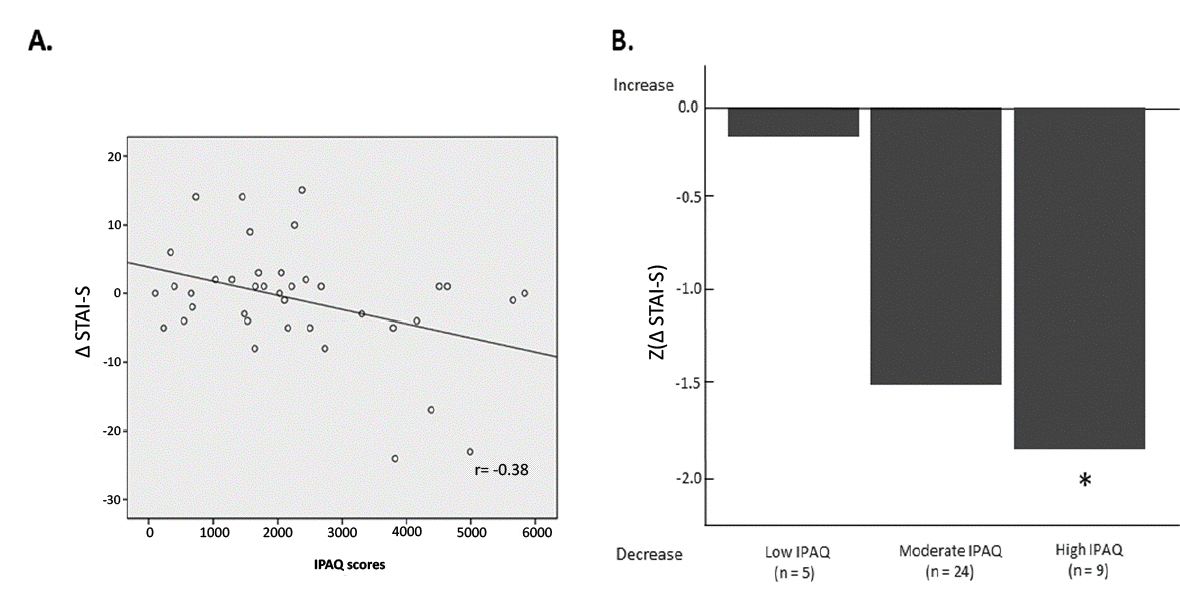


**Figure s4: Group-wise results of habitual physical activity, amygdala reactivity, and acute exercise-induced anxiolytic effect.**

**A.** The exercise-induced amygdala reactivity to explicit fear was correlated with IPAQ scores (*r* = -0.35, *p* = .029).

**B.** After identifying groups with low, moderate, and high IPAQ scores, the individual amygdala reactivity to explicit fear (EF–EN) was normalized and a non-parametric one-sample-*t* test (Wilcoxon signed rank tests) was performed separately for each group. The low IPAQ group (*n* = 5) had no significance (Z = -0.41, *P* = .67), whereas the moderate (*n* = 24) and high IPAQ (*n* = 9) groups achieved significant exercise-induced amygdala reactivity (Z = -1.7, *P* = .045, one-tailed; Z = -1.72, *P* = .043, one-tailed).

**C.** The exercise-induced amygdala reactivity to explicit fear was correlated with △STAI-S (*r* = 0.36, *p* = .026). Individuals with stronger exercise-induced amygdala reactivity reported more anxiety relief after exercise.
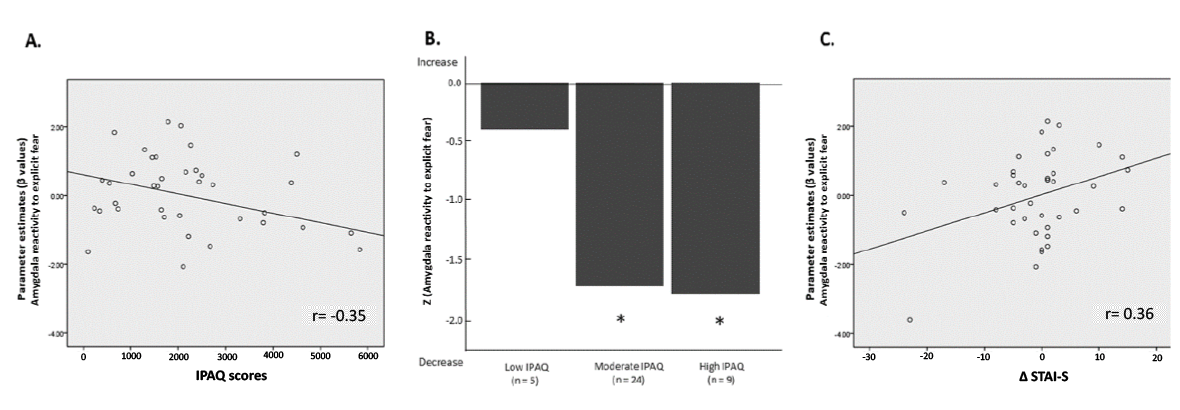

Supplement: Supplementary file 1 — Supplementary Materials [file 41598_2019_56226_MOESM1_ESM.doc]
